# Crusted Scabies

**DOI:** 10.1590/0037-8682-0199-2024

**Published:** 2024-09-02

**Authors:** Nurimar Conceição Fernandes, Carolina Piccinini Silva, Gabriel Castro Tavares

**Affiliations:** 1 Universidade Federal do Rio de Janeiro, Hospital Universitário Clementino Fraga Filho, Serviço de Dermatologia, Rio de Janeiro, RJ, Brasil.; 2 Universidade Federal do Rio de Janeiro, Hospital Universitário Clementino Fraga Filho, Serviço de Dermatologia, Programa de Residência Médica em Dermatologia, Rio de Janeiro, RJ, Brasil.

**FIGURE 1:**
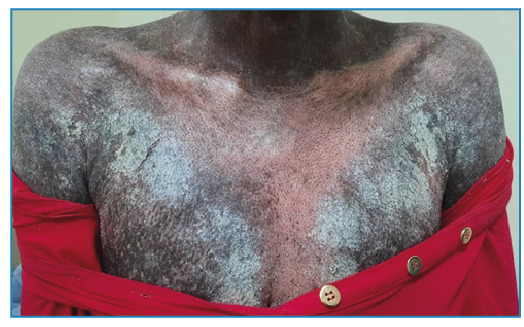
Patient showing diffuse grayish keratotic scales.

**FIGURE 2:**
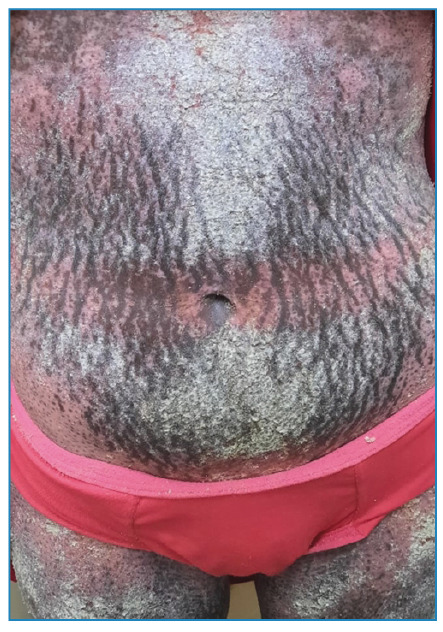
Grayish keratotic plaques on the abdomen with skin striae.

**FIGURE 3:**
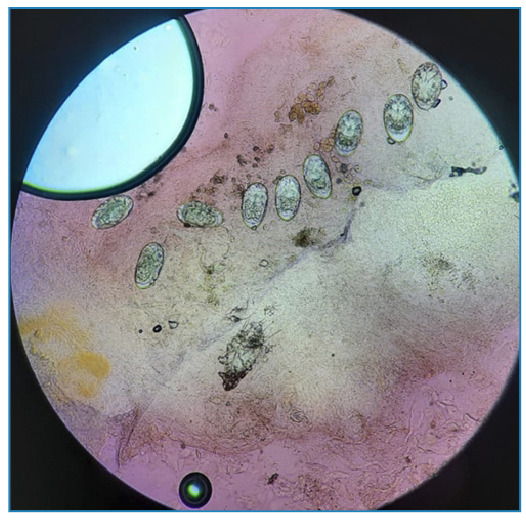
Potassium hydroxide scraping showing *Sarcoptes scabiei* mites.

A 25-year-old female from Rio de Janeiro presented with thick, gray, crusty keratotic scales on the skin throughout the body and mild itching at night (Figures 1 and 2). This immunocompromised, malnourished patient had manifested the disease for 1 month. Family members denied having similar signs and symptoms. She was hospitalized with single-room isolation and contact precautions. Microscopic examination with potassium hydroxide revealed mites and eggs of *Sarcoptes scabiei var. hominis* (Figure 3). Ivermectin (200 µg/kg) was prescribed once a week for 4 weeks, besides topical mineral oil twice daily. The patient died from skin secondary infection, sepsis, and advanced autoimmune disease. Crusted scabies (CS) is a rare clinical manifestation of scabies characterized by large crusted lesions and generalized thick grayish hyperkeratosis containing millions of highly contagious mites^1^ ^,^ ^2^. Although scabies are estimated to affect millions of people, no CS prevalence data exist globally or in Brazil. It spreads continuously through direct skin-to-skin contact with carriers or indirect contact with bedding and clothing. CS has been linked to a high mortality rate^1^. The most common cause of death is staphylococcal bacteremia. Ivermectin is a safe non-ovicidal drug, and in CS, it is given weekly depending on the severity and therapeutic response. Side effects include headache, nausea, dizziness, and gastrointestinal upset. Many side effects are thought to result from mite deaths rather than from the drug itself^3^. CS should be differentiated from psoriasis, seborrheic dermatitis, atopic dermatitis, and erythroderma. The grayish color of skin lesions is highly suggestive.
